# Complete radiological and biochemical remission at 26 months in a young adult with primary mediastinal yolk sac tumor: a case report and literature review on multimodal management

**DOI:** 10.1186/s13256-026-06553-3

**Published:** 2026-08-31

**Authors:** Yuanli You, Mingxing Yang, Xiuqing Wang, Wen Dong

**Affiliations:** Department of Respiratory Medicine, Hainan Cancer Hospital, Haikou, China

**Keywords:** Primary mediastinal yolk sac tumor, Multidisciplinary team, Complete radiological and biochemical remission at 26 months, Case report

## Abstract

**Background:**

Primary mediastinal yolk sac tumor (PMYST) is an extremely rare and highly malignant germ cell tumor that predominantly affects young males and is associated with a poor prognosis.

**Case presentation:**

We present the case of a 23-year-old non-smoking male diagnosed with stage III (cT4N0M0) PMYST who received comprehensive treatment guided by a multidisciplinary team (MDT). The treatment regimen included 5 cycles of neoadjuvant chemotherapy with BEP (bleomycin, etoposide, and cisplatin), followed by radical surgery, postoperative radiotherapy, and 4 cycles of adjuvant chemotherapy. Post-treatment, the patient's serum tumor markers normalized, with a partial radiological response (PR) and sustained biochemical remission. As of the latest follow-up in August 2025 (26 months after diagnosis), the patient remains in complete radiological and biochemical remission with no signs of recurrence.

**Conclusions:**

This case demonstrates that an intensive MDT-guided multimodal approach incorporating aggressive neoadjuvant chemotherapy, radical surgery, and consolidative sequential radiotherapy and chemotherapy may lead to encouraging outcomes, including the achievement of complete radiological and biochemical remission documented at 26 months. However, this case is more reflective of individualized treatment for a specific patient, and it also highlights the need for intensified surveillance beyond two years.

## Introduction

Yolk sac tumor (YST), also known as endodermal sinus tumor, is a highly malignant germ cell neoplasm that originates from primitive embryonic germ cells. It occurs most commonly in the gonads, with the mediastinum being the most frequent extragonadal site. Primary mediastinal yolk sac tumor (PMYST) is exceedingly rare, accounting for approximately 15.4% of all YSTs and only 1% of all primary mediastinal tumors [1]. It predominantly affects adolescent and young adult males aged 10–40 years. PMYST is characterized by aggressive behavior, rapid growth, frequent invasion of adjacent mediastinal structures (such as great vessels, pericardium, and the lungs) at diagnosis, and markedly elevated serum alpha-fetoprotein (AFP) levels [2, 3]. This report details the case of a young male with locally advanced (stage III) PMYST who achieved complete radiological and biochemical remission at 26 months through a multimodal treatment strategy guided by an MDT, including neoadjuvant chemotherapy, delayed surgery, and postoperative sequential radiotherapy and chemotherapy. This report aims to contribute to the understanding of PMYST management and to illustrate a potential treatment approach.

## Case description

A 23-year-old non-smoking male presented to our department with fever, cough, sputum production, and shortness of breath. Contrast-enhanced computed tomography (CT) revealed a large mass (approximately 13.5×12.3×7.9 cm) in the right anterior mediastinum. The mass invaded the adjacent brachiocephalic vein, with evidence of thrombus formation, and caused complete atelectasis of the right middle lobe. A malignant mediastinal germ cell tumor, likely YST, was suspected. Electronic bronchoscopy showed extrinsic compression and stenosis of the right middle and lower lobe bronchi. A CT-guided biopsy of the mediastinal mass was performed. Histopathological examination revealed tumor cells composed of complex, diverse epithelial cells forming a reticular pattern with variably sized spaces, along with papillary and tubular structures. Immunohistochemistry (IHC) was positive for SALL4, partially positive for PLAP and AFP, positive for panCK and CK19, positive for GPC-3, and negative for CD117, Oct-4, CD30, TTF-1, CK7, CD5, and CK5/6. Ki-67 was expressed in 90% of cells. HCG was negative (Figs. 1A–D). The morphological and IHC findings supported the diagnosis of a germ cell tumor, favoring YST. Systemic evaluation revealed no distant metastases. The final diagnosis was PMYST, stage III (cT4N0M0). Laboratory tests showed markedly elevated AFP>2000.00 ng/mL (normal: 0–8.78 ng/mL), elevated CEA 21.82 ng/mL (normal: 0–5 ng/mL), elevated CA19-9 62.93 U/mL (normal: 0–37 U/mL), and normal HCG 1.2 mIU/mL (normal: 0–5 mIU/mL). The patient's Eastern Cooperative Oncology Group (ECOG) performance status was 0 at presentation and remained 0 throughout the treatment course, indicating fully active, unrestricted functionality.

**Fig.1:**
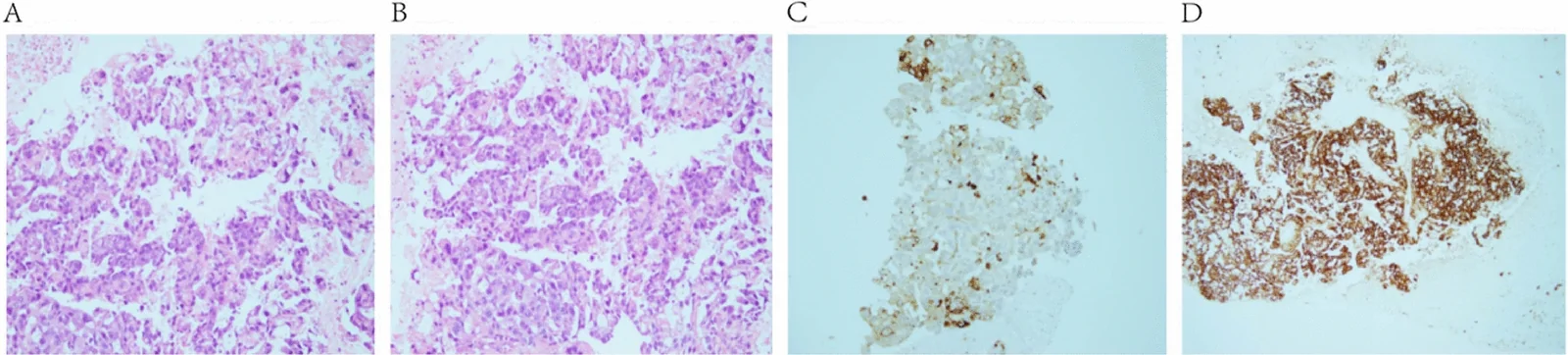
**A** Abnormal epithelioid cells with a high ratio of nucleoplasm to cytoplasm and thick chromatin were observed upon cytologic examination (H&E, 400 ×). **B** The tumor cells are arranged in a network and the cytoplasm is vacuolated (H&E, 400 ×). C Positive expression of AFP (400 ×). D Positive expression of GPC-3 (100 ×)

Given the locally advanced stage and close proximity of the tumor to the right middle/lower lobes and brachiocephalic vein, an MDT recommended neoadjuvant chemotherapy with planned delayed surgery. Given the patient's young age (23 years) and the known gonadotoxic effects of platinum‑based chemotherapy, fertility preservation was discussed with the patient prior to initiating chemotherapy. The patient underwent sperm cryopreservation before treatment. The patient received 5 cycles of BEP chemotherapy (bleomycin, etoposide, and cisplatin) from June to October 2023, with supportive care. Follow-up CT showed significant tumor reduction to 6.7 × 4.9 cm, indicating a partial response (PR). Serum AFP, CEA, and CA19-9 levels gradually normalized (AFP: 8.16 ng/mL; CEA: 4.95 ng/mL; CA19-9: 25.45 U/mL) (Fig. 2A–C).

**Fig.2:**
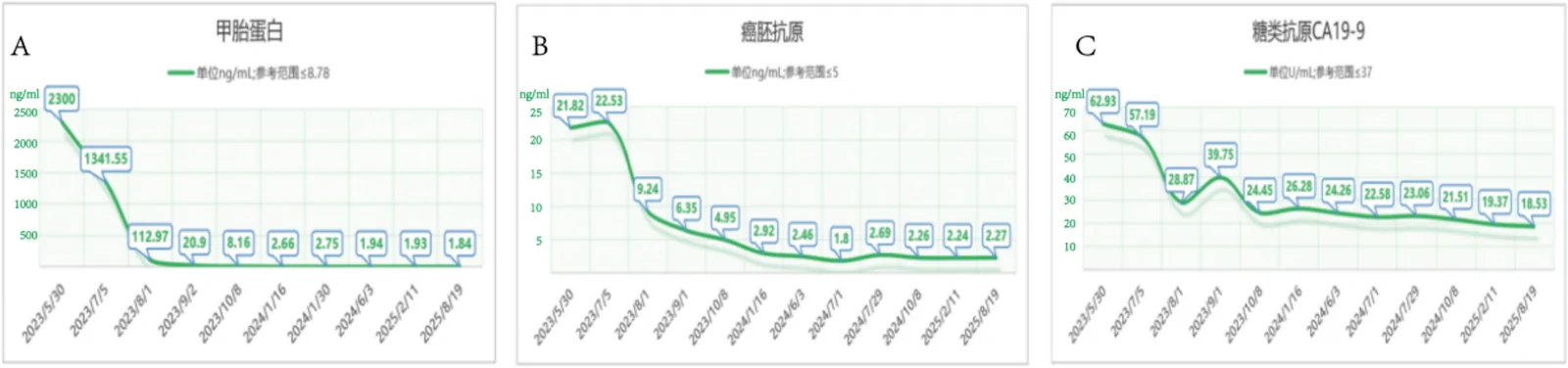
**A-C** The temporal course of AFP, CEA, and CA19-9 assessments during the treatment course from 2023 to 2025. *AFP* Alpha-fetoprotein, *CEA* Carcinoembryonic Antigen

Serum AFP half-life was calculated based on the decline from the pre-treatment level (2300.0 ng/mL) to the post-neoadjuvant chemotherapy level (8.16 ng/mL) over a 131-day interval (from May 30, 2023, to October 8, 2023). Using the formula t₁/₂ = 0.693 × Δt/ln(C₀/Cₜ), the estimated half-life was approximately 16.1 days. This exceeds the normal AFP half-life of 5–7 days. Our analysis suggests that this may be due to the sustained release of AFP from degenerated tissues, rather than active tumor proliferation. And in the subsequent follow-up process, the expression level of AFP remained normal, which confirmed sufficient treatment response.

A second MDT discussion recommended surgical resection with placement of intraoperative radiopaque clips for postoperative radiotherapy planning. On November 24, 2023, the patient underwent video-assisted thoracoscopic surgery (VATS) for extended resection of the anterior mediastinal tumor, including partial resection of the right middle and lower lobes and right phrenic nerve excision. The resected specimen measured 12×8×4 cm (Figs. 3A–B). Postoperative pathology revealed extensive necrosis with abundant histiocyte infiltration and scattered degenerated epithelial tumor cells, consistent with treated germ cell tumor changes. All margins were negative (R0 resection). These findings were consistent with a near-complete pathological response (near-pCR), rather than a complete pathological response (pCR), as scattered degenerated cells remained. No viable tumor cells were identified in the surgical margins or in the resected lymph nodes. IHC post-surgery was negative for AFP, GPC-3, SALL4, CK, CD117, CDX2, Hepatocyte, Oct-2, SOX-2, CK7, EMA, PLAP, Oct-4, CD30, and CK19. Ki-67 was 5% positive. While this marked reduction from 90 to 5% is notable, such post-treatment changes in proliferation marker expression should be interpreted cautiously, as chemotherapy-induced cellular changes can affect Ki-67 labeling. D2-40 was positive in stromal cells (Fig. 4A–D). The patient recovered well. A third MDT discussion recommended postoperative sequential radiotherapy and chemotherapy due to the initial vascular invasion and to minimize local recurrence.

**Fig.3:**
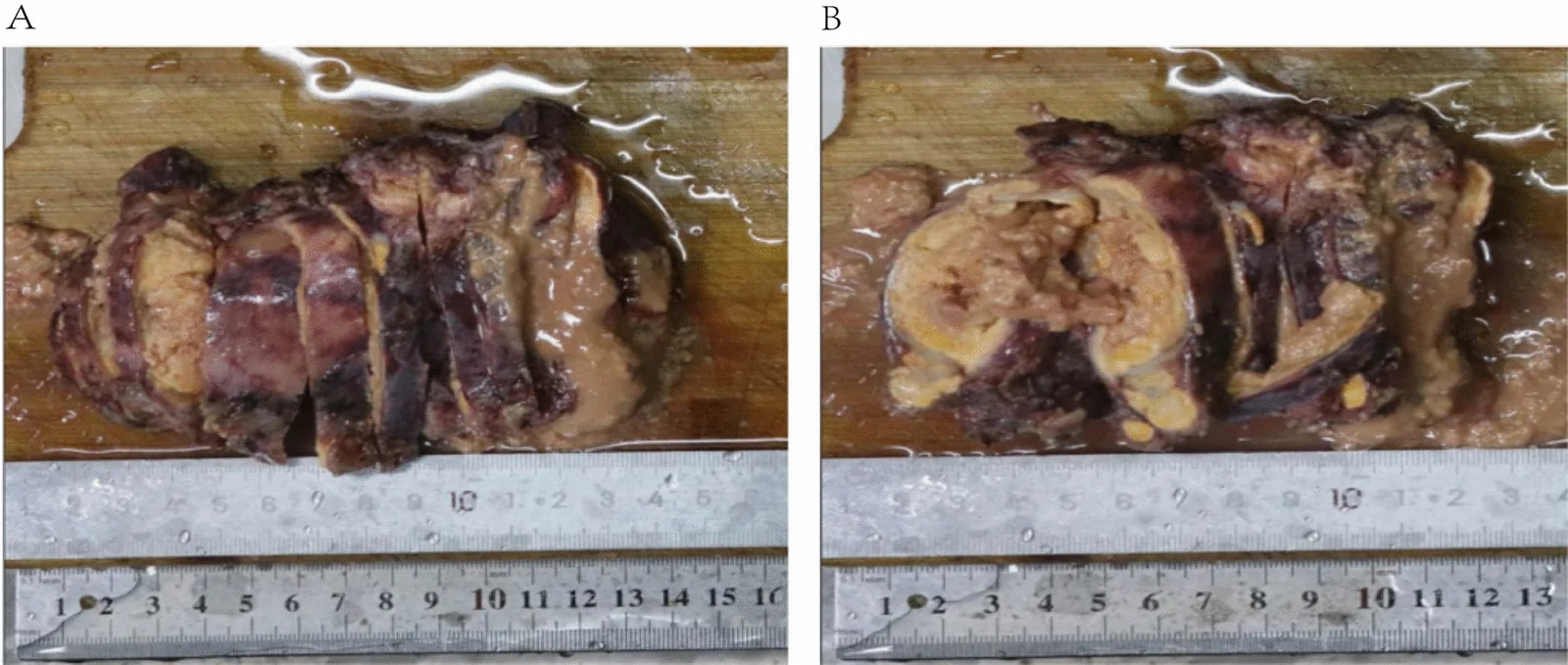
**A–B** Visual examination of surgical resection specimens: The outer layer of the tumor is covered by a capsule; The cut surface is gray yellow in color, with a medium texture and large areas of necrosis visible inside

**Fig.4:**
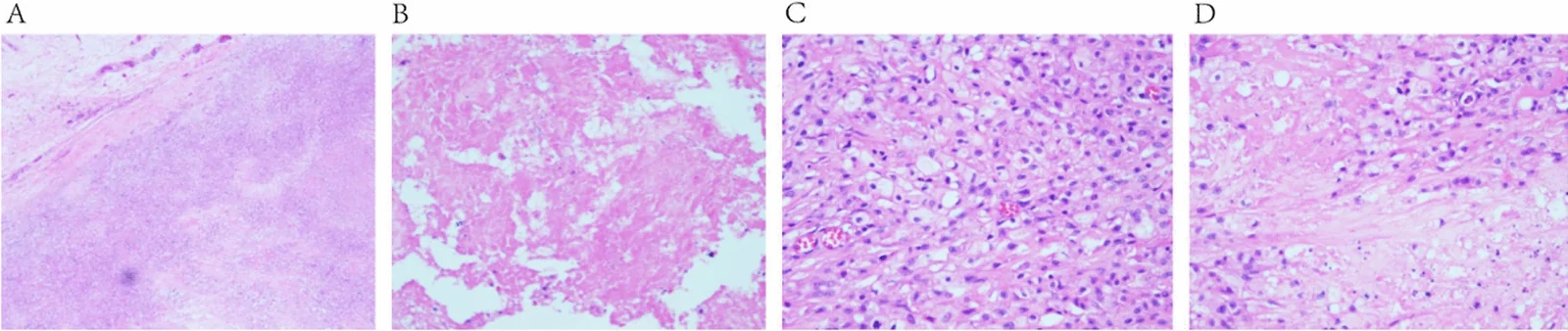
Postoperative histopathology and immunohistochemical staining. **A** Intact capsule. **B** Extensive necrotic tissues (H&E, 400 ×). **C** degenerated epithelial tumor cells (H&E, 400 ×). **D** necrotic tissues and degenerated epithelial tumor cells (H&E, 400 ×)

From January 25, 2024, the patient received postoperative radiotherapy to the mediastinum using intensity-modulated radiotherapy (IMRT) with a five-field coplanar technique. The clinical target volume (CTV) encompassed the postoperative tumor bed. The planning target volume (PTV) was generated by expanding the CTV with a 5-mm margin. The prescribed dose was 50 Gy in 25 fractions, delivered once daily, five fractions per week, Monday to Friday. Cone-beam CT (CBCT) was performed weekly for setup verification. Lung dose constraints were carefully optimized: V20 (volume of lung receiving ≥20 Gy) was kept below 28%, and mean lung dose (MLD) was maintained at <15 Gy, both meeting accepted tolerance limits. The heart and esophagus were also contoured and kept within tolerance. The patient tolerated the radiotherapy well, with no grade ≥2 pulmonary or esophageal toxicity observed during the treatment course.

This was followed by 4 cycles of adjuvant chemotherapy with etoposide and nedaplatin from April 23 to July 29, 2024. Adverse effects including myelosuppression and nausea/vomiting were managed supportively.

Treatment-related adverse events were monitored and graded according to the Common Terminology Criteria for Adverse Events (CTCAE) v5.0. During chemotherapy, the patient experienced grade 1 nausea and grade 2 myelosuppression, both of which can be controlled through supportive treatment without the need for reduction or delayed treatment. During radiotherapy, no grade ≥2 pulmonary or esophageal toxicity was observed. No grade ≥3 adverse events occurred throughout the entire treatment course.

Regular follow-ups were conducted. The latest follow-up was in August 2025, and the patient's overall survival (OS) had exceeded 2 years, reaching 26 months. The patient was in a state of continued complete radiological and biochemical remission with no evidence of recurrence. No long-term sequelae were reported by the patient at the 26-month follow-up. Specifically, no symptoms of bleomycin-induced pulmonary toxicity (e.g., dyspnea, dry cough, or exercise intolerance), cisplatin-related neuropathy or nephrotoxicity, or functional impairment from phrenic nerve resection (e.g., diaphragmatic elevation or respiratory compromise) were observed.

Representative CT images before and after neoadjuvant chemotherapy are shown in Fig. 5. The pre-treatment scan (Fig. 5B) demonstrated a 13.5×12.3×7.9 cm anterior mediastinal mass with brachiocephalic vein invasion and complete atelectasis of the right middle lobe. The post-chemotherapy scan (Fig. 5D) showed marked tumor shrinkage to 6.7×4.9 cm with partial re-expansion of the right middle lobe, confirming a partial response (PR) by RECIST v1.1 criteria.

**Fig.5:**
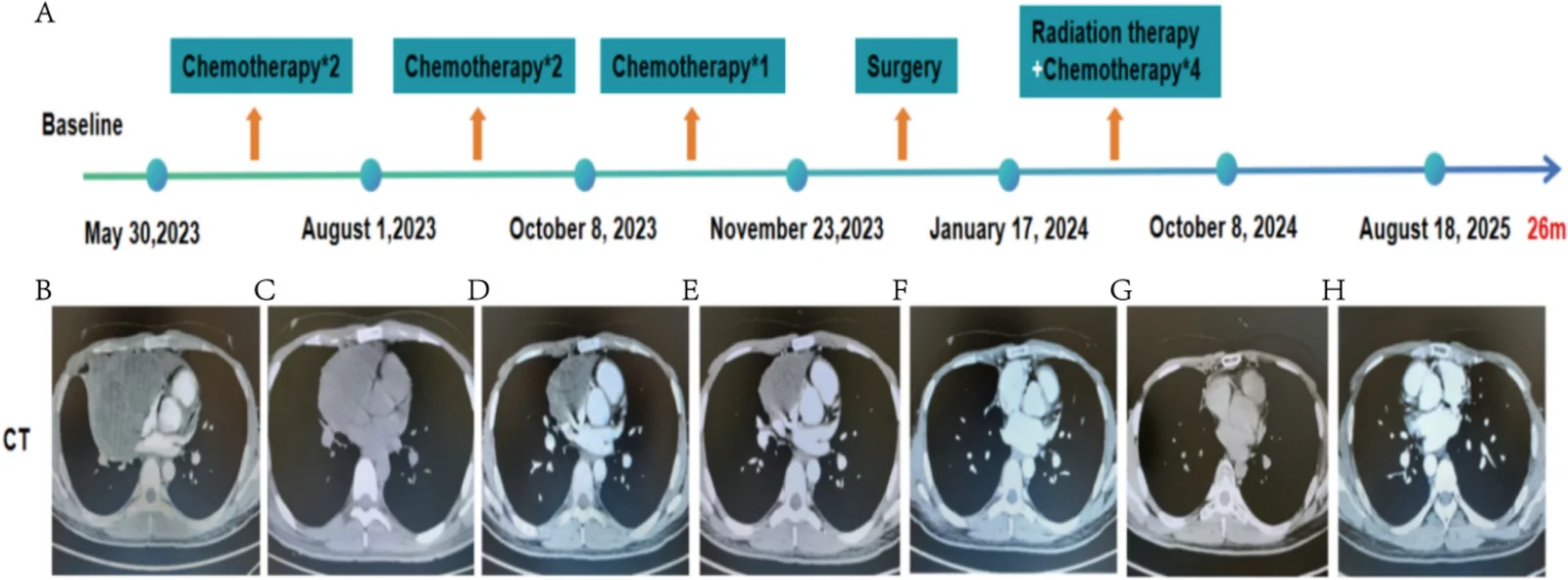
The temporal course of the treatment process and radiological evaluations from 2023 to 2025. At the time of this report, the patient's current overall survival is 26 months. The patient is currently off treatment and undergoing regular follow-up examinations. *CT* Computed Tomography

## Discussion

PMYST typically arises in the anterior mediastinum. These tumors are often large and frequently invade adjacent structures such as the trachea, great vessels, and pericardium, causing symptoms like chest pain and dyspnea. Rapid growth and early metastasis are hallmarks of PMYST. At diagnosis, metastatic spread (to lungs, liver, brain, bone) is common, making complete resection challenging and leading to a poor prognosis with high mortality and low 2-year survival rates [3]. The pathogenesis of PMYST remains unclear but may involve aberrant migration or arrest of germ cells during embryogenesis [4].

Diagnosis relies on characteristic histology (e.g., reticular, papillary, tubular patterns) and a distinctive IHC profile, integrated with clinical and radiological findings. This case met standard diagnostic criteria: (1) a young non-smoker male presenting with symptoms (fever, dyspnea) from a mediastinal mass; (2) CT showing a large anterior mediastinal mass with local invasion (brachiocephalic vein, lung), typical of PMYST; (3) histology showing characteristic patterns and IHC positivity for SALL4, PLAP (focal), AFP (focal), and GPC-3, while being negative for Oct-4, CD30, and HCG, effectively ruling out seminoma, choriocarcinoma, and primary lung adenocarcinoma [5]. Differential diagnoses include mediastinal seminoma (typically CD117+/Oct-4+ with normal AFP) and lung adenocarcinoma (often CK7+/TTF-1+, unlike this case). Metastatic YST from a gonadal primary was excluded via systemic imaging.

Elevated serum AFP levels (> 90% of cases) are a hallmark of PMYST. Since only YST cells among mediastinal tumors can produce AFP, AFP is considered a key diagnostic and prognostic biomarker [6]. In this case, AFP was >2000 ng/mL at diagnosis and normalized after neoadjuvant chemotherapy. Notably, CEA and CA19-9 were also mildly elevated at presentation and normalized with treatment. Several mechanisms may account for this observation. First, the bulky mediastinal mass may have caused biliary or hepatic congestion secondary to compression of adjacent structures. Cholestasis has been shown to play an important role in causing elevated CA19-9 levels, and elevated CA19-9 can occur even in the absence of hyperbilirubinemia [7]. Second, although PMYST typically exhibits yolk sac differentiation, the presence of glandular or epithelial components within the tumor may also contribute to CEA expression. Yolk sac tumors with somatic glandular patterns can reproduce developing intestinal, hepatic, or lung tissue and may express CEA [8]. However, given their lack of specificity, the clinical utility of CEA and CA19-9 in PMYST remains uncertain, and they should not replace AFP as the primary biomarker. Further studies are needed to validate their potential role as adjunctive markers in this rare tumor.

Due to its rarity, most of the existing literature is limited to case reports; there are no standardized staging or treatment guidelines for PMYST; the thymoma staging system is often adopted. Previous analyses, such as by Wu et al., indicate that long-term survivors (> 5 years) typically received multimodal therapy centered around surgery (e.g., chemotherapy + surgery ± radiotherapy) [3]. Given the proximity to vital structures, management is complex. There is no unified treatment regimen among domestic and international scholars currently. Common strategies include neoadjuvant chemotherapy (BEP or VIP regimens) followed by resection of residual disease and adjuvant chemotherapy, or surgical resection and postoperative chemotherapy [9]. Thus, MDT-guided multimodal therapy is crucial for success [10]. Neoadjuvant chemotherapy can downstage tumors, improve resectability, reduce surgical morbidity, and enhance overall survival. For our patient, who presented with stage III disease and a large invasive tumor, direct surgery carried extremely high risks and made achieving an R0 resection unlikely. We adopted the decision reached through MDT consensus and administered neoadjuvant chemotherapy first. After 5 cycles of standard BEP chemotherapy, resulting in a ~60% reduction in tumor size and normalization of tumor markers, achieving significant radiological and biological remission (PR, AFP normalization), and creating optimal conditions for successful R0 resection. This "chemotherapy-first" strategy has become the standard for locally advanced non-seminomatous germ cell tumors (NSGCTs), aiming to reduce tumor size, eliminate micrometastases, and improve the R0 resection rate. This case is consistent with the reported benefits of this approach, but this observation should not be interpreted as evidence of general treatment efficacy.

Beyond confirming the established multimodal treatment paradigm, this case report offers several specific clinical insights that are rarely documented collectively in the PMYST literature. First, it provides a detailed kinetic profile of tumor marker normalization, particularly the rarely reported parallel decline of CEA and CA19-9 alongside AFP. This observation raises the hypothesis that these markers might reflect epithelial or glandular components within the tumor, suggesting their potential as adjunctive biomarkers in AFP-producing tumors. Furthermore, our case illustrates the challenge of managing patients in the “gray zone” of near-pCR. The decision to intensify adjuvant therapy was not automatic; it was driven by the presence of pre-treatment vascular invasion and residual degenerated epithelial cells in the resected specimen—both of which raised our concern for recurrence. We discuss this individualized approach explicitly in the paper. Third, the report contributes practical safety data on the tolerability of a high cumulative platinum dose (9 cycles) and sequential thoracic radiation following bleomycin exposure, with detailed documentation of toxicity monitoring. These provide actionable reference points for clinicians encountering similar complex cases.

Radical resection of all residual disease is considered a cornerstone of treatment for preventing local recurrence and achieving cure, even after a good chemotherapy response [2]. Surgery is the cornerstone of treatment for mediastinal YST. Extended resections, even involving invaded organs, can yield favorable outcomes. Yang et al. reported significantly improved 5-year survival rates for patients undergoing surgery compared to chemotherapy or radiotherapy alone (60.3% vs. 35.8%, P<0.001) [2]. In this case, extended radical resection was performed after chemotherapy, and with placement of intraoperative radiopaque clips for postoperative radiotherapy planning. Although pathology indicated extensive treatment effect (near-complete pathological response), surgery aimed to remove potentially resistant cells and achieve local control.

The role of adjuvant therapy post-resection remains debated. It is generally considered for patients with advanced initial stage (III/IV), residual viable tumor post-chemotherapy (even if pathology mainly indicates necrosis), or invasion of critical structures such as large blood vessels or nerves. Adjuvant BEP chemotherapy is a standard option [3, 11, 12]. Sakaguchi M et al. reported a case of postoperative recurrence of PMYST that was successfully treated with radiotherapy, suggesting that radiotherapy in the salvage setting may be an alternative option for patients with chemotherapy-resistant or recurrent disease [13]. Qin L et al. reported a patient with lung metastasis who received chemotherapy combined with immunotherapy, resulting in significant tumor regression [14]. This suggests that immunotherapy may become a new treatment modality for yolk sac tumors, which requires further research and analysis. However, it is important to emphasize that this evidence pertains to salvage radiotherapy rather than adjuvant radiotherapy, and thus does not provide direct support for the routine use of adjuvant radiotherapy after R0 resection.

Our patient received postoperative radiotherapy and 4 cycles of etoposide/nedaplatin chemotherapy, mainly based on the following considerations: (1) The initial tumor invaded the brachiocephalic vein with thrombus formation, posing a risk of intravascular minimal residual disease; (2) Although the postoperative pathology showed extensive necrosis and IHC negativity for all markers, the presence of scattered degenerated epithelial tumor cells (near-pCR, rather than pCR) still indicated a potential risk of recurrence, particularly given the pre-treatment vascular invasion; (3) Nedaplatin, a second-generation platinum analog, has been reported to have a more favorable renal and gastrointestinal toxicity profile compared to cisplatin in certain clinical settings, although its evidence in germ cell tumors is less robust. This substitution was made on an individualized basis to reduce the cumulative risk of nephrotoxicity and severe gastrointestinal intolerance, allowing the patient to complete the planned adjuvant therapy. We acknowledge that this represents an institutional practice variation rather than a standard recommendation. (4) The patient is young (23 years old), with good physical condition throughout the entire treatment period (ECOG score of 0), and can tolerate intensive treatment under close monitoring.

Given the intensive nature of this treatment regimen—comprising 5 cycles of neoadjuvant BEP, lung resection, mediastinal radiotherapy, and 4 cycles of adjuvant platinum-based chemotherapy—the patient was closely monitored for cumulative toxicity throughout the treatment course. Renal function (serum creatinine, estimated glomerular filtration rate), hepatic function, and peripheral neurologic symptoms were assessed before each chemotherapy cycle. Pulmonary function was monitored by clinical assessment (symptoms of dyspnea, cough, exercise tolerance) before and after radiotherapy, with no grade ≥2 toxicity observed. We emphasize that this intensified adjuvant approach was tailored to this specific high-risk case and should not be generalized to all PMYST patients. We have discussed this decision as an individualized, risk-adapted strategy rather than a standard recommendation.

PMYST carries a poor prognosis, with most recurrences occurring within 1–2 years. Disease-free survival beyond 2 years significantly increases the likelihood of cure, underscoring the importance of long-term follow-up. This patient, despite advanced initial stage, demonstrated sensitivity to BEP chemotherapy, achieved R0 resection, and maintained normalized tumor markers, indicating a favorable prognosis. However, it must be acknowledged that although a 26-month disease-free survival is encouraging and exceeds the typical 1–2 year recurrence window for PMYST. Long-term cure (traditionally defined as≥5 years of disease-free survival) still requires continued surveillance to confirm.

The patient is currently on a structured surveillance protocol. Serum tumor markers (AFP, CEA, CA19-9) are measured every 3 months for the first 2 years, and every 6 months thereafter. Contrast-enhanced CT of the chest and abdomen is performed every 6 months for the first 2 years, and annually thereafter. Brain MRI and bone scintigraphy are performed annually to screen for distant metastases, given the potential for extramediastinal spread. This protocol will continue for at least 5 years post-treatment.

In conclusion, this case illustrates the successful management of advanced PMYST in a young adult through an MDT-guided multimodal strategy, with the patient achieving complete radiological and biochemical remission at 26 months. It highlights several key points: (1) Integrated diagnosis using histology, IHC, and serum markers is essential. (2) Neoadjuvant chemotherapy is effective for downstaging and facilitating R0 resection. (3) Radical surgery remains pivotal, even after a good response to chemotherapy. (4) The postoperative adjuvant radiotherapy and chemotherapy were administered as an individualized management decision, based on the patient’s initial vascular invasion and near‑complete pathological response; however, the efficacy of this adjuvant regimen cannot be determined from this single case report. (5) complete radiological and biochemical remission at 26 months can be achieved in individual cases with aggressive, comprehensive treatment. This report provides a case-based reference and may offer encouragement for clinicians managing this rare and complex malignancy.

## Data Availability

The raw data used and/or analyzed during the current research period can be obtained from the first author upon reasonable request.
